# Sleep and smartphone use: Within and between-person relationships from an objective longitudinal smartphone and wearable data donation study

**DOI:** 10.1371/journal.pdig.0001232

**Published:** 2026-02-13

**Authors:** Paulien Decorte, Karolien Poels, Cedric Vuye, Jonas Lembrechts, Ablenya Barros, Karolien Couscheir, Gert-Jan De Bruijn

**Affiliations:** 1 Marketing and Supply Chain Management, School of Business and Economics, Maastricht University, Maastricht, The Netherlands; 2 Department of Communication Studies, Social Sciences, University of Antwerp, Antwerp, Belgium; 3 Department of Construction Engineering, Applied Engineering, University of Antwerp, Antwerp, Belgium; 4 Ecology and Biodiversity, Environmental Biology, University of Utrecht, Utrecht, The Netherlands; University of Wurzburg: Julius-Maximilians-Universitat Wurzburg, GERMANY

## Abstract

Poor sleep is common and detrimental to health. Smartphone use is often noted as a sleep disruptor, but evidence remains limited and inconsistent. This necessitates research focused on objective, longitudinal designs, as well as analytical approaches that can reveal lagged and reciprocal relationships that capture within- and between-person effects. To address these gaps, the current study investigated within- and between-person lagged and reciprocal effects of sleep duration and smartphone use of 68 participants through longitudinal and objective data donated from iPhones and Apple Watches across 14 consecutive days. Apple Watches objectively measured total sleep and sleep stage durations (REM, core, and deep sleep), while iPhones assessed total smartphone use duration and in-bed smartphone use. Two Dynamic Structural Equation Models (DSEMs), one with total sleep and one with sleep broken down into three sleep stages, were conducted. At the within-person level, more total smartphone use increased same-day in-bed smartphone use, *β* = .25 (95% CI .20, .31), which in turn led to more same-day overall sleep, *β* = .08 (95% CI .02, .14). Additionally, results indicated stable between-person habits, with strong day-to-day associations for each variable with its own next-day value, *β* = .53-.82 (95% CI .47, .88). Findings contradict the perspective of smartphones as sleep disruptors, despite leaving open whether this added sleep means poorer rest or a real benefit of in-bed smartphone use. Furthermore, the strength of the between-person results emphasizes the importance of habits in this relationship. In studying day-to-day smartphone use and sleep, these findings provide nuanced empirical insights supporting health and policy recommendations regarding smartphone use and sleep hygiene.

## Author summary

Poor sleep is widespread and linked to many health problems. Smartphone use is often blamed for making sleep worse. However, past findings have been mixed, and many studies rely on self-reports or short-term designs. In this study, we examined how daily smartphone use and sleep relate to each other over time using objective data. We analyzed two weeks of real-world data from iPhones and Apple Watches worn by 68 adults. This allowed us to track how much people used their phones, how often they used them in bed, and how long they slept each night, including different types of sleep.

We found that on days when people used their smartphones more overall, they were also more likely to use them in bed. Surprisingly, greater in-bed smartphone use was linked to slightly longer sleep duration the same night. These patterns suggest that smartphones may not always reduce sleep time, as is commonly assumed. At the same time, we observed strong and stable individual habits: people who tended to sleep or use their phones more on one day were likely to do so again the next day. Overall, our findings highlight the importance of daily habits and suggest a more nuanced relationship between smartphone use and sleep than simple disruption.

## Introduction

Sleep is vital to both mental and physical health [1,2], though many people struggle with insufficient sleep [3]. Excessive smartphone use is frequently cited as a contributor to poor sleep quality [4,5], with evidence linking it to fatigue and disrupted sleep even when it is used with the intention of aiding sleep [6,7]. However, recent studies have begun to challenge the notion that smartphone use is uniformly harmful to sleep [8], revealing mixed findings and suggesting a more complex relationship than previously assumed. Notably, some research indicates that sleep disturbances may actually lead to increased smartphone use [9], pointing to potential reciprocal effects that have yet to be fully understood.

To address these ambiguities, the current study takes a novel approach by investigating reciprocal relationships between smartphone use and sleep over time, examining how each may influence the other both within and between individuals. This approach allows inferences of both time-varying and habitual ties between sleep and smartphone use [10]. The current study harnesses objective*,* longitudinal data – which is scarce in this area [8,11,12] – captured through smartphones and wearables. The Apple Watch, for instance, has demonstrated sleep measurement validity against gold standards [13,14], making it suitable to objectively measure sleep. As this device is increasingly used by individuals to track their own health behaviors [15,16] - reflecting a larger growing habit of digital health tracking in everyday life [17] - adopting it in this study allows for high ecological validity as well as a detailed operationalization of both sleep and smartphone use. This includes specific in-bed usage patterns and distinctions between sleep stages, which can further improve the empirical evidence of this relationship [11,18]. Analytically, the study applies Dynamic Structural Equation Models (DSEMs) to disentangle the complex interplay between smartphone use and sleep. This method enables robust modelling of both lagged and reciprocal effects across and within days—capturing how sleep may influence next-day smartphone use, and how smartphone use may affect sleep later that same night [19].

By integrating cutting-edge objective measurement with sophisticated longitudinal modelling, this research provides a uniquely detailed and dynamic view of the bidirectional relationship between sleep and smartphone use. The findings will help clarify previous mixed results and inform more precise health and policy recommendations around smartphone use and sleep hygiene.

## Methods

### Ethics statement

All participants provided written informed consent in Qualtrics. This study was approved by the Ethics Committee for the Social Sciences and Humanities at the University of Antwerp on March 6^th^ 2024 (SHW_2024_10_2).

### Design

This work adopted a data donation design [20], where people provide data from their iPhone and Apple Watch (AW) over 14 consecutive days. Sleep data, including total sleep duration and sleep phases (REM, core, deep), were collected via the Apple Watch. Smartphone use data, including total usage and in-bed usage, were obtained through iPhone Screen Time screenshots.

### Participants

Data were available from a subsample of participants in a citizen science study in Belgium (De Oorzaak). Through data donation methods, these participants provided objective sensor-based data derived from their AW (WatchOS ≥ 9) and iPhone (iOS ≥ 16) across a six-week period by uploading XML (AW) and JPG (iPhone) files through Qualtrics. Belnet Filesender was used to securely transfer files larger than the 30MB allowed by Qualtrics. Participants' iPhone Screen Time (ST) screenshots spanned 14 consecutive days, since collecting 42 days of screenshots was too demanding. The current dataset is therefore limited to 14 days.

After expressing interest, they provided informed consent and shared their sociodemographic information (e.g., gender, age, education, income). Data collection spanned March-June 2024. Participants were not compensated.

### Procedure

Participants were instructed to export their data from their iPhone and AW and upload this securely through Qualtrics. For iPhone ST data, participants took screenshots of their ST app for the past 14 days. Since it is not possible to filter AW data by time or measure beforehand – all data is exported – the researchers pre-processed the data keeping only project-relevant measures (sleep, heart rate, noise exposure) within the data collection timeframe and deleted identifying data before analysis. The full protocol adhering to STROBE guidelines is available on OSF: https://osf.io/yxmv5

### Variables

#### Sleep.

AW Sleep app data (WatchOS ≥ 9) consists of different sleep stages: *Awake*, *REM*, *Core* (N1 and N2 stages), and *Deep* (N3 stage or slow-wave sleep) [21], widely recognized in sleep process research [22]. REM sleep is not seen as restful and consists of dreaming, resembling brain activity similar to being awake [22]. N1-N3 stages are considered non-rapid eye movement (NREM) sleep, taking up to 75% of sleep and progressively leading to deeper sleep [22]. Of those, N1 (5%) is the lightest sleep stage lasting around 1–5 minutes per sleep cycle. N2 (45%) is deeper and important for memory consolidation, indicated by drops in body temperature and heart rate. N3 (25%) is the deepest NREM sleep, also known as slow-wave sleep, where the body repairs and people are most difficultly awakened [22]. Though these various sleep stages are marked by various physiological signals, including muscle tone, heart rate, temperature, brain activity, and blood oxygen [22], the AW mainly captures movement and respiratory motion via an Apple-developed classifier algorithm from the AW’s triaxial accelerometer signals [21]. Apple’s own sleep validation resulted in 97.9% sensitivity (sleep classified correctly) and 75% specificity (awake classified correctly) [21]. Other studies also reported high accuracy and sensitivity [13], including compared to reference polysomnography devices [14]. We included the three sleep stages separately (*REM, Deep, Core*) and calculated their sum to obtain participants’ *total sleep* per day.

#### Total smartphone use.

Screenshots participants took of their daily ST reports, a native iPhone app [23], provided data on total daily iPhone use (hours and minutes), hourly use duration, and the three most-used app durations. We manually extracted these values from the screenshots and entered them into a database, coding total smartphone use (TSU) based on the recorded total daily duration. While the main author coded the screenshots, a second researcher (KP) independently verified 10% of the dataset (*n* = 96 screenshots), demonstrating very good agreement between coders.

#### Smartphone use in bed.

In-bed times can be inferred from participants’ pre-set iPhone sleep schedule reflecting their sleep/wake times via “Sleep Focus” that participants can manually turn on/off [24]. However, to more accurately determine participants’ in-bed intervals, we derived in-bed times from AW accelerometry (StepCount) [25] rather than relying solely on iPhone’s preset Sleep Focus schedule. AW step count accelerometer data were processed by identifying sustained periods of minimal movement during typical evening, nighttime, and early-morning hours. Long inactivity gaps and epochs with very low step counts were used to infer transitions into and out of bed, with brief and isolated low-activity moments classified as wake-after-sleep-onset rather than full exits from bed, which were inferred from activity across several subsequent periods following these gaps. This accelerometry-based classification allowed us to reconstruct in-bed periods for each participant. We then coded their hourly iPhone use duration via ST screenshots during these in-bed stages and summed this smartphone use in bed (SUiB).

### Data cleaning

76 participants shared AW data and ST screenshots. We deleted *n* = 8 participants with ≥7 days of missing data for either sleep or ST, leaving *N* = 68 participants with 952 total data points across 14 consecutive days for analysis.

Apple Watch data and iPhone screenshots may have contained personal information. To ensure privacy, data were filtered and pseudonymized. An R script anonymized Apple Watch XML files (e.g., renaming device labels) and retained only variables of interest, linking them to participant ID numbers. All other raw data were deleted. Screenshots, which could include participant names, were converted into anonymous numerical datasets (e.g., usage hours, app types), and the original images were deleted.

### Data analysis

We estimated two-level Dynamic Structural Equation Models (DSEMs) using R 4.3.2 and the *brms* package to assess the relationship between smartphone use and sleep, accounting for both within and between-person differences, as well as lagged and reciprocal effects. We tested two models. In the first, we included total sleep time, TSU duration, and SUiB duration in seconds. In the second, we replaced total sleep with the three sleep stages to see which are more strongly affected by smartphone use or vice-versa. Multiple imputation with 5 models was adopted for missing values, pooling the results. We calculated lagged variables (*t*-1) for lagged and autoregressive effects but kept the effects of TSU and SUiB towards all sleep variables current, as well as the effect of TSU on SUiB. Variables were grand mean centered to extract person-specific means (between-person component) and individual deviations from those means across time (within-person component) [26].

DSEM incorporates Bayesian estimation with uninformative priors [27], using two Markov Chain Monte Carlo (MCMC) simulations. Through this estimation, if the value of 0 falls within the 95% credible interval (CI) for a specific estimate, we conclude that it is non-significant [28]. Models were run with 5,000 iterations, with standardized parameter estimates (*β*) interpreted, representing means of posterior distributions (OSF https://osf.io/yxmv5). The potential scale reduction (PSR) factor or R-hat [29] determined model convergence, with R-hat < 1.01 indicating adequate model convergence [30].

To assess whether extreme observations influenced model estimates, we conducted posterior predictive checks for the mean of each outcome. For each imputed dataset, we simulated replicated data from the posterior predictive distribution and calculated the proportion of simulations in which the replicated mean exceeded the observed mean (posterior predictive “p-value”). Values near 0.5 indicate that the observed mean is typical under the model [31]. Across outcomes, the models reproduced the means well (0.51–0.68). This indicates that the outliers did not materially affect the central tendency of the modeled outcomes.

## Results

### Descriptives

Participants had a mean age of 43.76 (*SD* = 13.63). Over half were men (60.29%), employed full-time (57.35%), and had a net income of>€4000/month (52.95%). The large majority had pursued higher education (94.12%). Table 1 lists the sample demographics.

**Table 1:** Sample characteristics (N = 68).

Demographic Variable	Value
**Age - M (SD)**	43.76 (13.63)
**Gender - n (%)**	
Male	41 (60.29%)
Female	27 (39.71%)
**Employment - n (%)**	
Full-time employee	39 (57.35%)
Part-time employee	6 (8.82%)
Self-employed	10 (14.71%)
Retired	6 (8.82%)
Incapacitated for work	4 (5.88%)
Student	1 (1.47%)
Other	2 (2.94%)
**Education - n (%)**	
Higher secondary education	4 (5.88%)
Undergraduate higher education (bachelor)	32 (47.06%)
Graduate education (master, PhD)	32 (47.06%)
**Income - n (%)**	
€1001-€2000 per month	3 (4.41%)
€2001-€3000 per month	14 (20.59%)
€3001-€4000 per month	9 (13.24%)
€4001-€5000 per month	12 (17.65%)
€5001-€6000 per month	12 (17.65%)
> €6000 per month	12 (17.65%)
Prefer not to answer	6 (8.82%)

**Fig 1:**
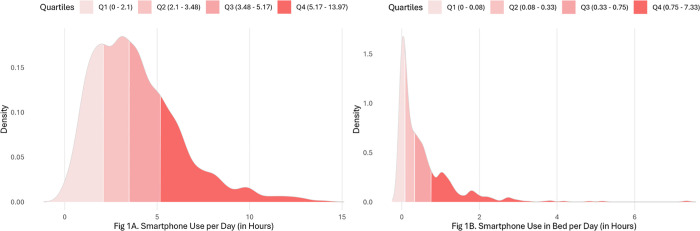
Density distribution of participants’ daily smartphone use duration (A) in total and (B) in bed.

Participants’ TSU ranged from 0-13.97 hours/day (*M* = 3.85, *SD* = 2.34), their SUiB from 0-7.3 hours/day (*M* = .54, *SD* = .73). Fig 1A-1B depict the density distributions of these variables.

**Fig 2:**
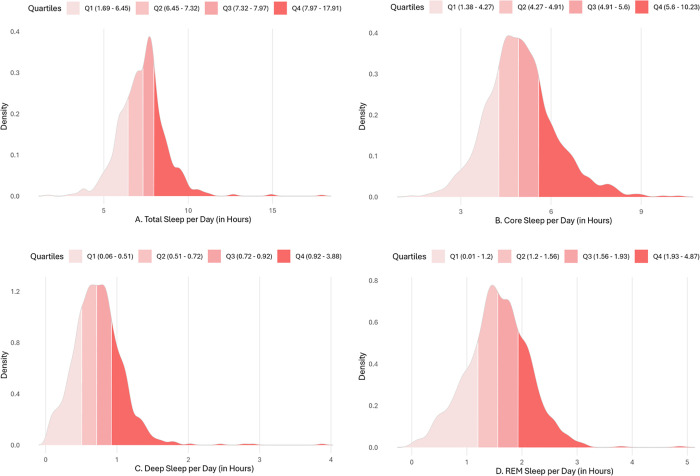
Density distribution of participants’ daily (A) total (B) core, (C) deep, and (D) REM sleep.

Participants’ total daily sleep ranged between 1.69-17.91 hours daily (*M* = 7.30, *SD* = 1.38). Participants spent 1.38-10.23 hours per day in core sleep (*M* = 4.98, *SD* = 1.16), .06-3.88 hours in deep sleep (M *=* .73, *SD* = .35), and .01-4.87 hours in REM sleep daily (*M* = 1.55, *SD* = .57). Fig 2A-2D present density distributions of total sleep and sleep stages.

### Model results

After 5000 iterations, the R-hat dropped below 1.01, indicating excellent model convergence for both models [30]. The standardized estimates and CIs are presented in Table 2 (Model 1) and Table 3 (Model 2). No multicollinearity issues arose, with Variance Inflation Factor (VIF) values in both models between 1.00-2.58, considering those above 3–5 to be problematic [32].

**Table 2:** Model 1 standardized parameter estimates (*β*) and 95%-CI.

	Total Sleep_t_		TSU_t_		SUiB_t_	
	*β*	95%-CI	*β*	95%-CI	*β*	95%-CI
*Within-Person*						
Total Sleep_*t-1*_	-.16*	[-.22;-.10]	-.05*	[-.09;-.01]	.01	[-.05;.02]
TSU_*t*_	-.02	[-.08;.05]	-.00_*t-1*_	[-.04;.04]	.25*	[.20;.31]
SUiB_*t*_	.08*	[.02;.14]	.10*_t-1_	[.05;.14]	.01_*t-1*_	[-.05;.07]
*Between-Person*						
Total Sleep_*t-1*_	.53*	[.47;.59]	.00	[-.03;.04]	.01	[-.05;.06]
TSU_*t*_	.01	[-.08;.09]	.82*_t-1_	[.76;.88]	-.02	[-.10;.05]
SUiB_*t*_	.00	[-.08;.09]	.01_*t-1*_	[-.05;.07]	.63*_t-1_	[.55;.71]

Estimates with 0 outside the 95% CI are significant [28], bolded with an asterisk.

**Table 3:** Model 2 standardized parameter estimates (*β*) and 95%-CI.

	TSU_t_		SUiB_t_		Core_t_		Deep_t_		REM_t_	
	*β*	95%-CI	*β*	95%-CI	*β*	95%-CI	*β*	95%-CI	*β*	95%-CI
*Within-Person*										
TSU_*t*_	-.00_*t-1*_	[-0.05; 0.04]	.25*	[.20;.31]	-.03	[-.10;.04]	.01	[-.04;.07]	.02	[-.05;.08]
SUiB_*t*_	.10*_t-1_	[.05; 0.14]	.01_*t-1*_	[-.05;.07]	.06	[-.00;.12]	.03	[-.03;.11]	.04	[-.02;.10]
Core_*t-1*_	-.05	[-.09;.00]	-.02	[-.08;.04]	-.05	[-.11;.02]	-.07*	[-.14;-.01]	-.10*	[-.16;-.04]
Deep_*t-1*_	.00	[-.04;.04]	.03	[-.03;.09]	-.03	[-.10;.03]	-.15*	[-.21;-.09]	-.02	[-.08;.03]
REM_*t-1*_	-.01	[-.06;.03]	-.04	[-.09;.02]	-.03	[-.11;.05]	.01	[-.05;.07]	-.06	[-.12;-.00]
*Between-Person*										
TSU_*t*_	.82*_t-1_	[.76; 0.88]	-.02	[-.11;.06]	.00	[-.08;.09]	.00	[-.08;.08]	.01	[-.07;.09]
SUiB_*t*_	.01_*t-1*_	[-.05;.08]	.63*_t-1_	[.55;.71]	.00	[-.08;.09]	.00	[-.08;.09]	.00	[-.09;.08]
Core_*t-1*_	.00	[-.04;.04]	.01	[-.05;.07]	.58*	[.51;.64]	-.03	[-.09;.03]	.00	[-.06;.06]
Deep_*t-1*_	.00	[-.05;.05]	-.01	[-.07;.06]	-.01	[-.08;.06]	.56*	[.49;.63]	.02	[-.05;.09]
REM_*t-1*_	.00	[-.04;.05]	.00	[-.06;.07]	.00	[-.07;.07]	.01	[-.06;.07]	.56*	[.49;.63]

Estimates with 0 outside the 95% CI are significant [28], bolded with an asterisk.

It should be noted that in Model 2, the inclusion of sleep phases (REM, core, deep sleep) reduced its predictive accuracy. A leave-one-out cross-validation (LOO) model comparison demonstrated a log predictive density (ELPD) of -2152.4 (*SD* = 49.8) for Model 2 compared to Model 1. This suggests that Model 1 without sleep stages has a substantially better predictive accuracy. Findings from Model 2 should therefore be interpreted cautiously.

#### Within-person.

**Fig 3:**
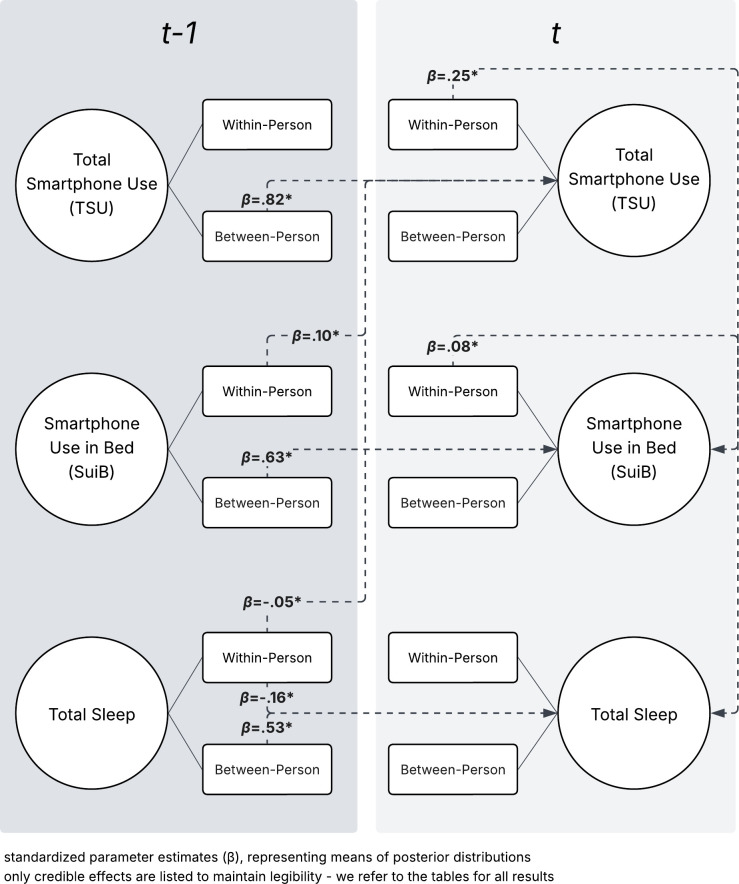
Diagram of Model 1 significant effects.

In Model 1 (Fig 3), more previous-day total sleep led to significantly less next-day total sleep (*β* = -.16). Greater TSU led to more same-day SUiB (*β* = .25), which in turn increased same-day total sleep (*β* = .08) and next-day total smartphone use (*β* = .10) - a cyclical effect. This showcases a day-to-day positive effect of smartphone use in bed on sleep, decreasing total smartphone use the next day (*β* = -.05).

**Fig 4:**
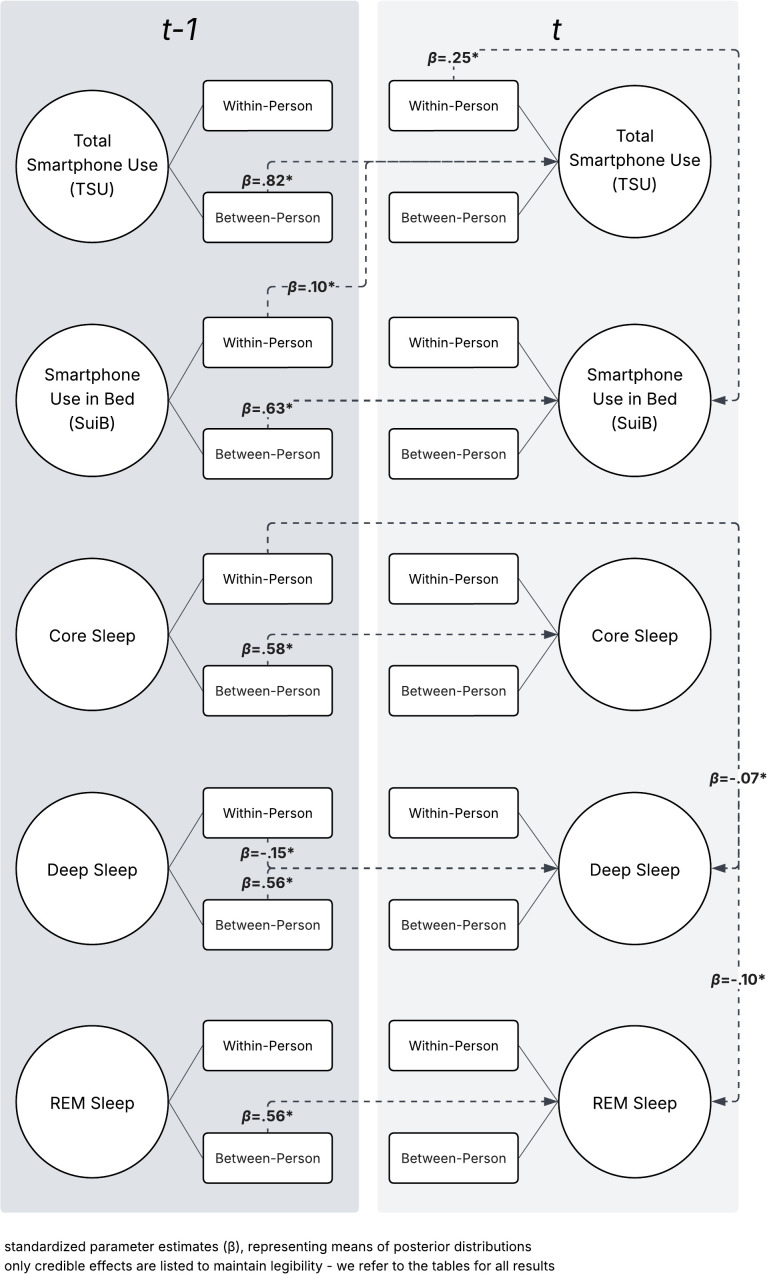
Diagram of Model 2 significant effects.

Some within-person sleep and smartphone behaviors led to decreased sleep duration in Model 2 (Fig 4). Previous-day core sleep reduced next-day deep (*β* = -.07) and REM sleep (*β* = -.10). Previous-day deep sleep led to less the next day (*β* = -.15). The positive association between same-day TSU and SUiB remained (*β* = .25), with a cyclical effect remaining where previous-day SUiB increased next-day TSU (*β* = .10) . Unlike Model 1, there was no significant positive relationship between SUiB and specific sleep stages, though they lean in a positive direction.

#### Between-person.

Significant positive between-person associations emerged between previous-day and next-day total sleep (*β* = .53), TSU (*β* = .82), and SUiB (*β* = .63) in Model 1. These results contrast the daily within-person fluctuations for this model: though previous-day and next-day sleep have a negative relationship within people, between-person results suggest that those who sleep more continue to do so.

In Model 2, the significant positive between-person associations between previous-day and next-day TSU (*β* = .82), and SUiB (*β* = .63) remained, along with positive associations between previous-day and next-day core (*β* = .58), deep (*β* = .56), and REM sleep (*β* = .56). We find similar contrasts here when comparing with the within-person results for this model: daily within-person shifts and between-person habits do not align.

## Discussion

This study investigated the relationship between smartphone use and sleep, overall and across sleep stages considering between- and within-person effects based on objective data from participants’ AWs and iPhones. The first DSEM model found significant positive effects of in-bed smartphone use on sleep. Such effects were not found for total smartphone use. More smartphone use also increased in-bed smartphone use in both models. At the between-person level, both previous-day sleep and smartphone use are strongly associated with the same next-day behaviors, demonstrating behavioral habits. Subsequent paragraphs will discuss these results in more detail.

Descriptive results regarding sleep and smartphone use largely align with the literature. Participants' sleep averaged 7–8 hours/day (Fig 3), which is recommended and associated with healthy sleep [33]. On AWs, core sleep comprises stages N1-N2 [21], which account for 50% of total sleep together [22], where the current results account for more, around 70% (Fig 3). REM and deep sleep (N3) each account for 25% of total sleep [22]. This study’s results reflect this for REM, but not for deep sleep (11%; Fig 3), which is a key difference considering this stage’s role in body repair and regrowth [22]. Participants’ TSU use also aligns with local results [34], though such results for SUiB are not available.

The first models’ within-person dynamics revealed that as individuals used their smartphone more in bed, they slept more overall the same day. Though these relationships are not significant regarding specific sleep stages in the second model, they also lean in a positive direction. This does not align with evidence that smartphones are problematic for sleep [4,7,35]. These results, along with the absence of significant effects of total smartphone use, demonstrate the importance of increased granularity in investigating smartphone use and sleep by focusing on in-bed smartphone use and sleep stages [7]. A cyclical relationship seemed to emerge, where greater total smartphone use led to greater in-bed use, which increased total sleep, in turn decreasing total smartphone use the next day. There is also a reinforcing effect between total smartphone use and in-bed use, where the increased in-bed use due to more total use also leads to more total use the next day. Taken together, this suggests that in-bed smartphone use, reinforced by total smartphone use, may contribute to increased sleep duration over time. Although in-bed use directly increases next-day total smartphone use, its sleep-prolonging effect may counteract this by reducing phone use across days, reflecting a truly dynamic relationship in which in-bed use can ultimately lead to longer sleep and stabilize overall smartphone use. However, it is important to note that longer sleep does not necessarily imply better sleep, where this increased sleep may indicate that in-bed smartphone use requires more sleep over time since people are less well-rested. In sum, despite this positive relationship between in-bed smartphone use and sleep, it is not yet clear whether this represents a beneficial or sleep supportive relationship.

Both models revealed negative relationships between previous-day and next-day sleep. This may be explained by sleep duration’s effect on fatigue [36], such that increased sleep may reduce fatigue, leading to less next-day sleep. Older adults, like the current sample, spend less time asleep, but more time in N1 [37] and N2 [22] or core sleep, which may further explain their effects of reducing next-day sleep stage durations.

Though significant, the within-subjects parameter estimates were small, suggesting that these behaviors do not show large day-to-day fluctuations. The between-person results confirm this, demonstrating habitual strength of smartphone use and sleep [18]: frequent in-bed smartphone use tends to persist, for instance. These habits can strengthen within-person patterns, impacting sleep and smartphone use long-term [18]. Overall, while there are significant within-person daily shifts to consider in the relationship between smartphone use and sleep, between-person habits seem to be stronger predictors of future sleep and smartphone use.

This study demonstrates the efficacy of integrating objective, longitudinal data collection with dynamic statistical modelling. Meaningful and temporally nuanced associations between sleep and smartphone use were identified within a relatively modest sample of 68 healthy participants over a 14-day period, underscoring the methodological strength and scalability of this approach. Furthermore, in populations characterized by more stable or extreme patterns—such as individuals with chronic sleep difficulties or excessive smartphone use—these associations may emerge more strongly, highlighting the potential of this method for future targeted research.

These findings make key contributions to the literature on sleep and smartphone use by adopting a novel longitudinal and objective data donation design, alongside analytically separating between- and within-person effects. To our knowledge, this is the first study in this domain to apply such a design, gleaning novel and called-for insights [18] regarding how smartphone use affects sleep as well as vice-versa, and which parts of sleep are affected by what kind of smartphone use. The current results contrast existing evidence that smartphone use negatively impacts sleep [4,7,35], where in-bed smartphone use may actually lead to increased sleep over time – though closely linked to overall usage habits and with a remaining question on whether this longer sleep reflects poorer rest or a true benefit resulting from in-bed smartphone use. Given the strong habitual patterns observed in both sleep and smartphone use, future interventions and policy efforts should consider targeting these routines, with special attention to in-bed use that may increase sleep over time. Overall, these findings help clarify mixed evidence in the field, revealing that smartphone use is not merely detrimental to sleep [8] and supporting the presence cyclical effects between smartphone use and sleep [9].

### Limitations and avenues for future research

This novel approach involves some limitations. Findings are based on a convenience sample , yielding mostly affluent and well-educated participants and limiting generalizability. The current sample size also restricts meaningful demographic comparisons, such as gender, regarding smartphone use and sleep. Future work can build on these observations by including representative samples or focusing on people actively struggling with sleep and/or with consistent smartphone use in bed to investigate this relationship further. Furthermore, we sampled owners of AWs and iPhones, limiting this study to participants with the necessary means. To approach a more inclusive sample, future work should provide such devices to participants.

Model comparison indicated that Model 1, which did not include sleep stages, had substantially better predictive accuracy than Model 2, which did. Findings from Model 2 should therefore be interpreted with caution. Additionally, while Model 2 did not reveal significant associations between in-bed smartphone use and sleep stages, an effect was observed for total sleep. This suggests that a larger or more detailed dataset may be needed to develop a better predictive model incorporating sleep stages in their relationship with (in-bed) smartphone use.

Promising methodological inclusions also remain to be adopted. First, we did not consider the content consumed on participants’ smartphones. Investigating this content consumed in bed can detail media use mechanisms and their effects on sleep [9]. This also enables research on media-multitasking and sleep [18], and on the effects of sleeping aid content [38]. Furthermore, observing awake times on the AW can yield more specific data on nighttime awakenings and subsequent smartphone use, shedding additional light on how and when smartphones are used as sleeping aids [18].

Finally, we reflect on the data donation design. First, while AWs are promising and show adequate validation for research [13], they do not replace gold standards like polysomnography [13]. Results are not clinical and should be interpreted considering that there may be errors, while carrying empirical value by studying this relationship objectively and longitudinally in a natural setting.

## Conclusion

While smartphones are often seen as detrimental to sleep, evidence is mixed, suggesting a reciprocal relationship. This study used novel methods to investigate this complex interaction via data donated from participants’ iPhones and AWs, yielding longitudinal, objective insights. We modeled temporal relationships considering lagged and reciprocal effects between and within individuals. Results demonstrated within-person fluctuations and reciprocal effects of sleep and smartphone use: increased in-bed smartphone use led to more same-day sleep. More total smartphone use increased same-day in-bed smartphone use, which in turn increased overall sleep. Both previous-day sleep and smartphone use behaviors were strongly associated with those same behaviors the next, demonstrating habitual between-person patterns. Findings contradict the view of smartphones as sleep disruptors, suggesting that the timing of use – particularly in-bed use – and dynamic smartphone–sleep interactions may increase sleep duration. However, additional research is warranted to clarify whether this effect reflects smartphone use that supports sleep, or rather a need for more sleep due to a lack of restorative sleep resulting from in-bed smartphone use. A focus on specific smartphone use timing and between-person habits may be key in promoting compounding sleep-improving effects of smartphone use.
